# Supplementary material for: TXNIP Regulates Natural Killer Cell-Mediated Innate Immunity by Inhibiting IFN-γ Production during Bacterial Infection
Source: Int J Mol Sci. 2020 Dec 14;21(24):9499. doi: 10.3390/ijms21249499 (PMC7765025; doi:10.3390/ijms21249499)
Supplement: Supplementary file 1 [file ijms-21-09499-s001.pdf]

SUPPLEMENTAL INFORMATION

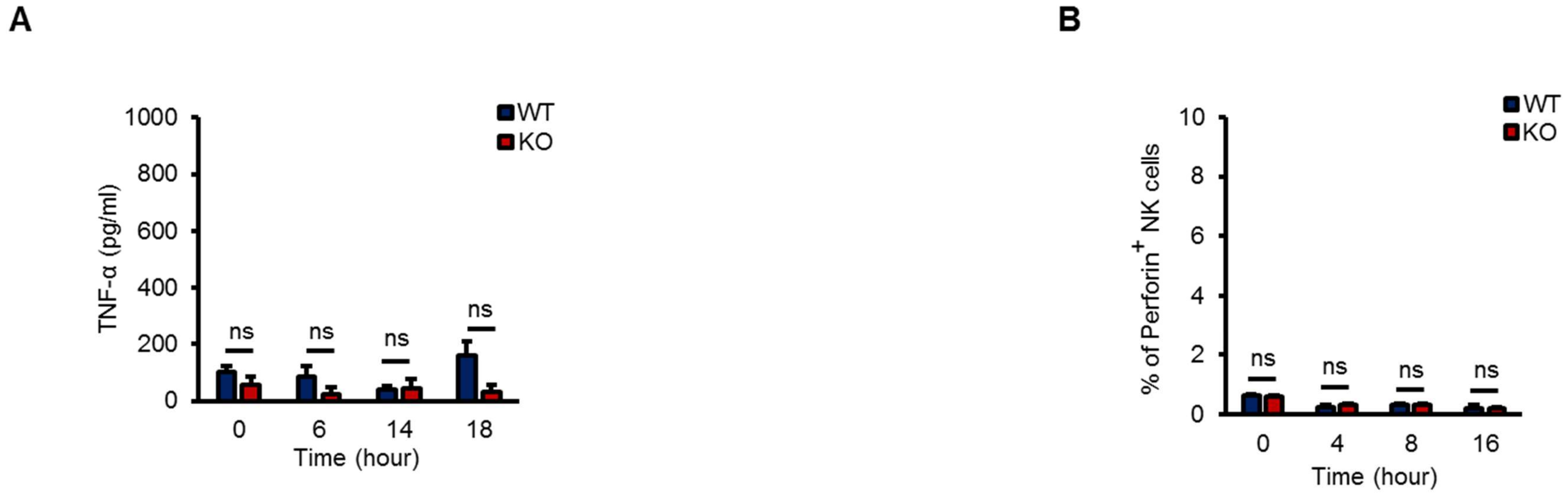

Figure S1. NK cells are not activated for the production of TNF-α and Perforin by Pam stimulation. WT and KO NK cells were cultured at 1 x 10^6 cells per well in 24-well plate and stimulated by Pam (1μg/ml) for 4h, 8h and 16h. **(A)** The concentration of TNF-α. **(B)** Frequency of Perforin positive cells of NK cells harvested from each wells. These experiments were independently repeated three times, and data are mean ± SD (n=3). Statistical significance was determined using Student's t tests. ns (not significant).

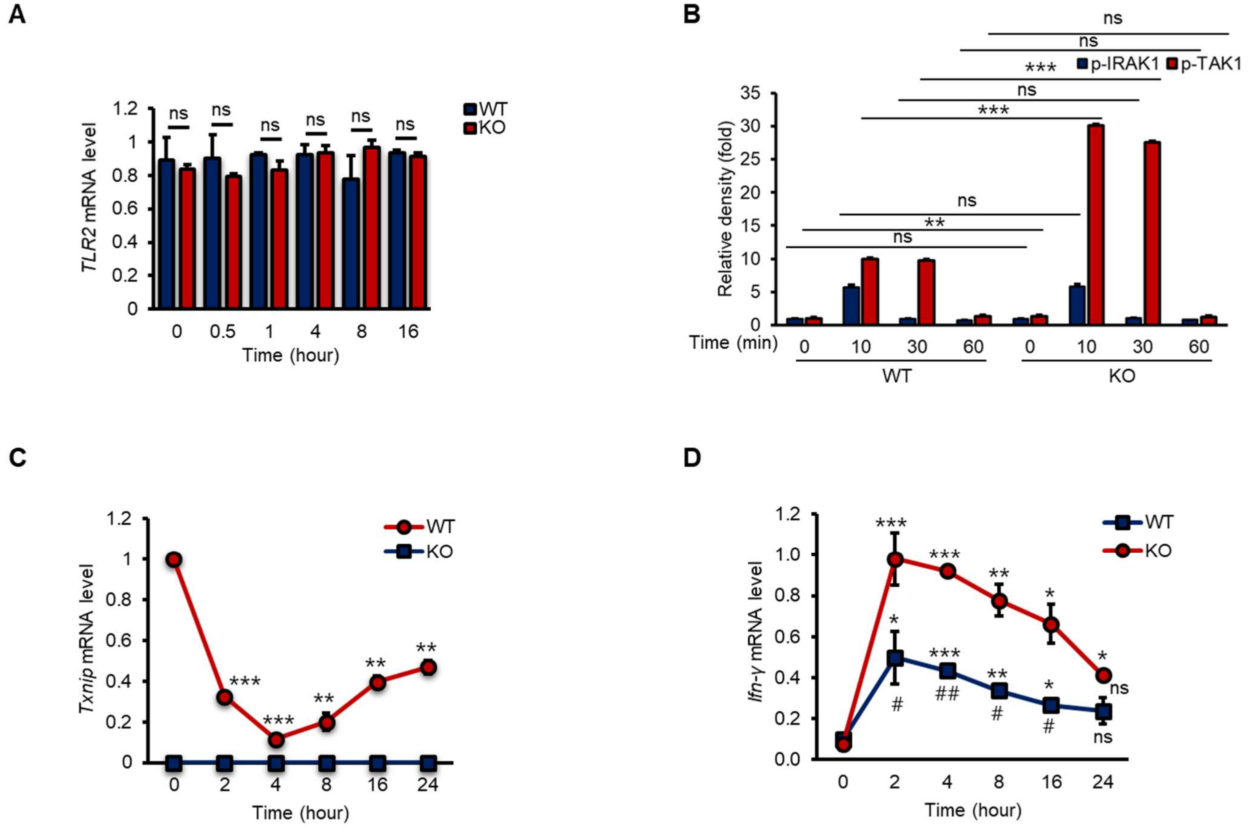

Figure S2. Regulation of gene expression in NK cells by Pam stimulation. **(A)** Quantitative real-time PCR results for TLR2 in WT or KO NK cells stimulated by Pam (1μg/ml) for 0-16h. **(B)** Densitometric analysis of p-IRAK and p-TAK1**.** The expression of Txnip **(C)** and Ifn-γ **(D)** in WT or KO NK cells stimulated by Pam (1μg/ml) for indicated time points. **p* compared to same genotype mice and #*p* WT mice versus KO mice. These experiments were independently repeated three times, and data are mean ± SD (n=3). Statistical significance was determined using Student's t tests. */#*p* < 0.05, **/##*p* < 0.01, ****p* < 0.001, ns (not significant).

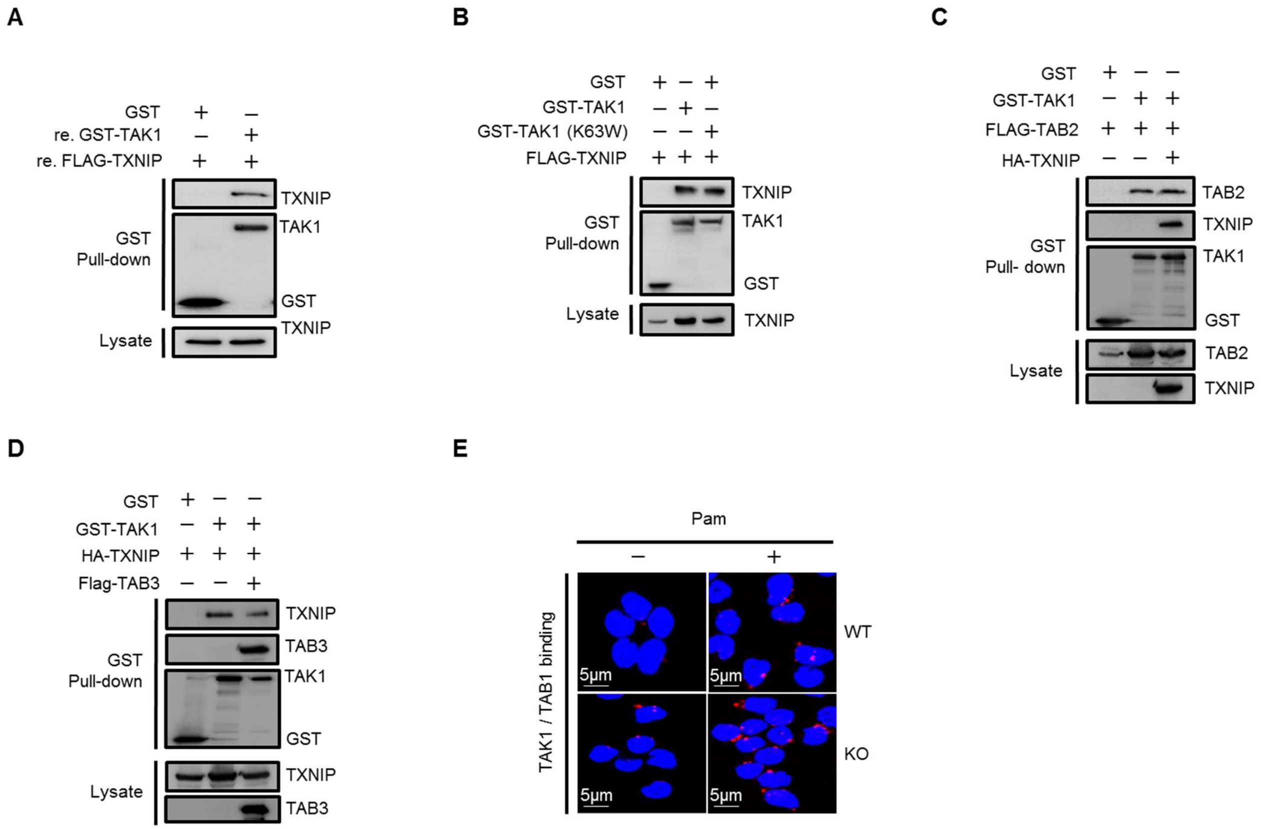

Figure S3. GST pull-down assays. **(A)** Purified recombinant TXNIP directly interacts with recombinant TAK1. We put two recombinant proteins (re.FLAG-TXNIP and re.GST-TAK1) together for 1h and the protein complexes were analyzed using GST pull-down assay. **(B)** Kinase activity of TAK1 is not important for the complex formation between TXNIP and TAK1. TXNIP does not compete with TAB2 **(C)** or TAB3 **(D)** for the complex formation with TAK1. Data are representative of three independent experiments. **(E)** *In situ* PLA images for TAK1-TAB1 complex in WT and KO NK cells following Pam (1μg/ml) treatment for 15min.

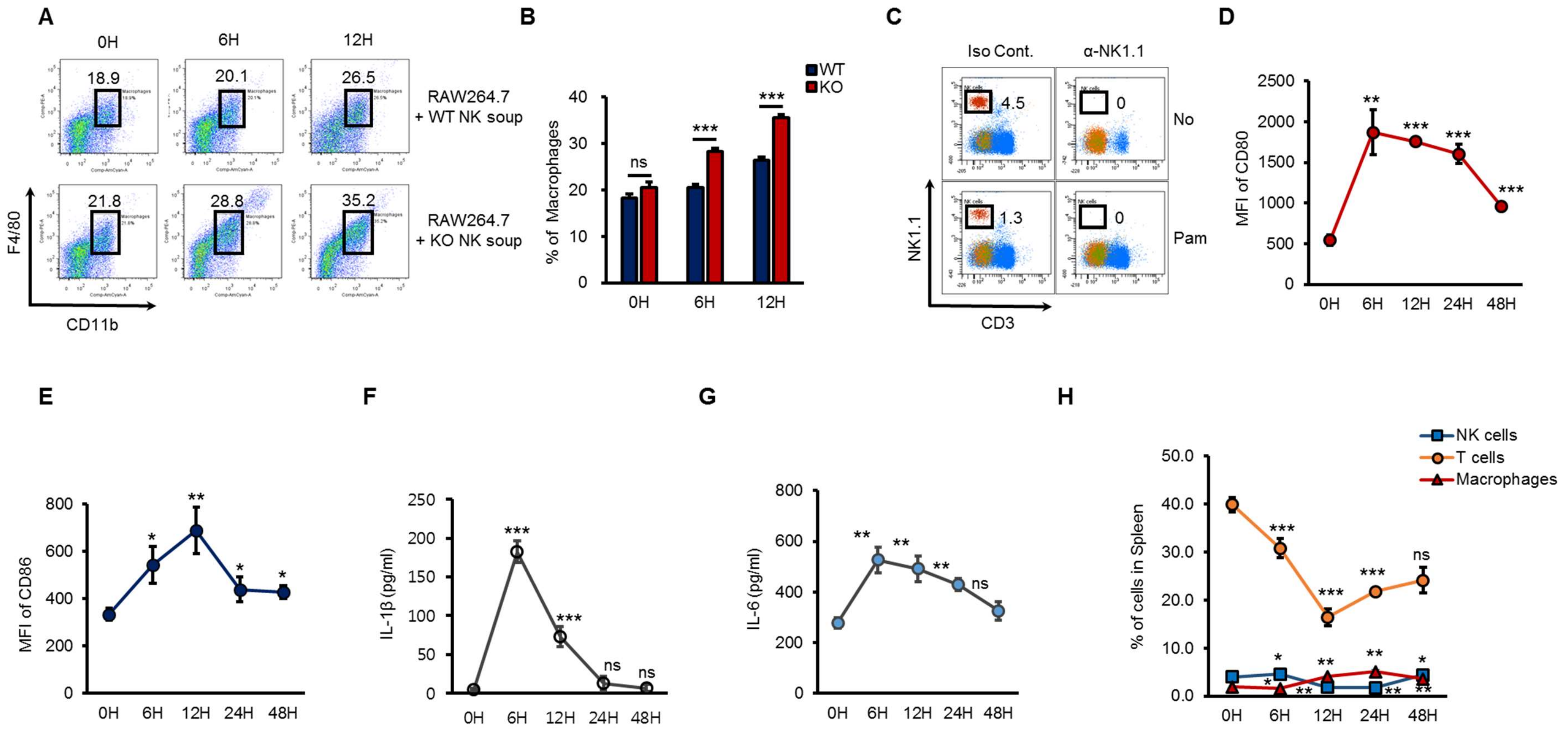

Figure S4. Activation of macrophages by NK cells *in vitro* and *in vivo*. **(A and B)** Frequency of CD11b$^+$F4/80$^+$ cells in RAW264.7 cells. WT and KO NK cells were stimulated by Pam (1μg/ml) for 16h and then RAW264.7 cells cultured in the soup from WT or KO NK cells culture (n=3). Repeated three times. **(C)** Representative dot plots of CD3$^-$NK1.1$^+$ cells in spleen of control or NK cell-depleted mice at 12h after Pam (2μg/g) injection (n=5). Repeated three times. **(D-H)** Pam induces the inflammation responses in vivo. C57BL6/J mice (n=8) were intraperitoneally injected by Pam (2μg/g) and sacrificed at 0, 6, 12, 24 and 48h respectively. The expression of CD80 **(D)** and CD86 **(E)** on the surface of macrophages in spleen was determined by flow cytometry. The

concentration of IL-1β **(F)** and IL-6 **(G)** was measured by ELISA in mice serum. **(H)** Frequency of NK cells, T cells and macrophages was analyzed by flow cytometry in mice spleen. Repeated three times. Data are mean ± SD. Statistical significance was determined using Student's t tests. *$p < 0.05$, **$p < 0.01$, ***$p < 0.001$, ns (not significant).

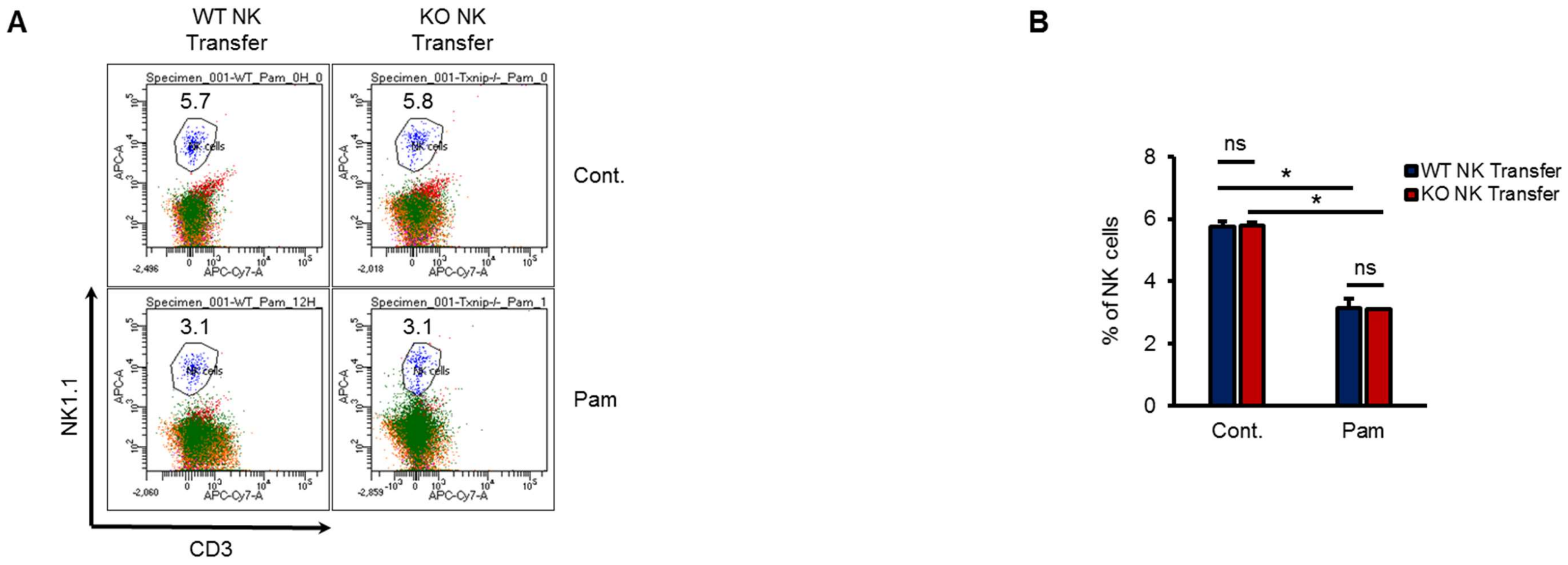

Figure S5. Frequency of adoptive transferred NK cells in Rag2$^{-/-}$ x Il2rg$^{-/-}$ mice. **(A and B)** WT or KO NK cells were equally transferred into Rag2$^{-/-}$ x Il2rg$^{-/-}$ mice and were maintained equally after Pam (2μg/g) stimulation. Representative dot plots of NK (CD3^{-}NK1.1^{+}) cells **(A)** and frequency of NK cells **(B)** in the spleen at 12h after stimulation. These experiment were independently repeated three times and data are mean ± SD (n=5). Statistical significance was determined using Student's t tests. *$p < 0.05$, ns (not significant).

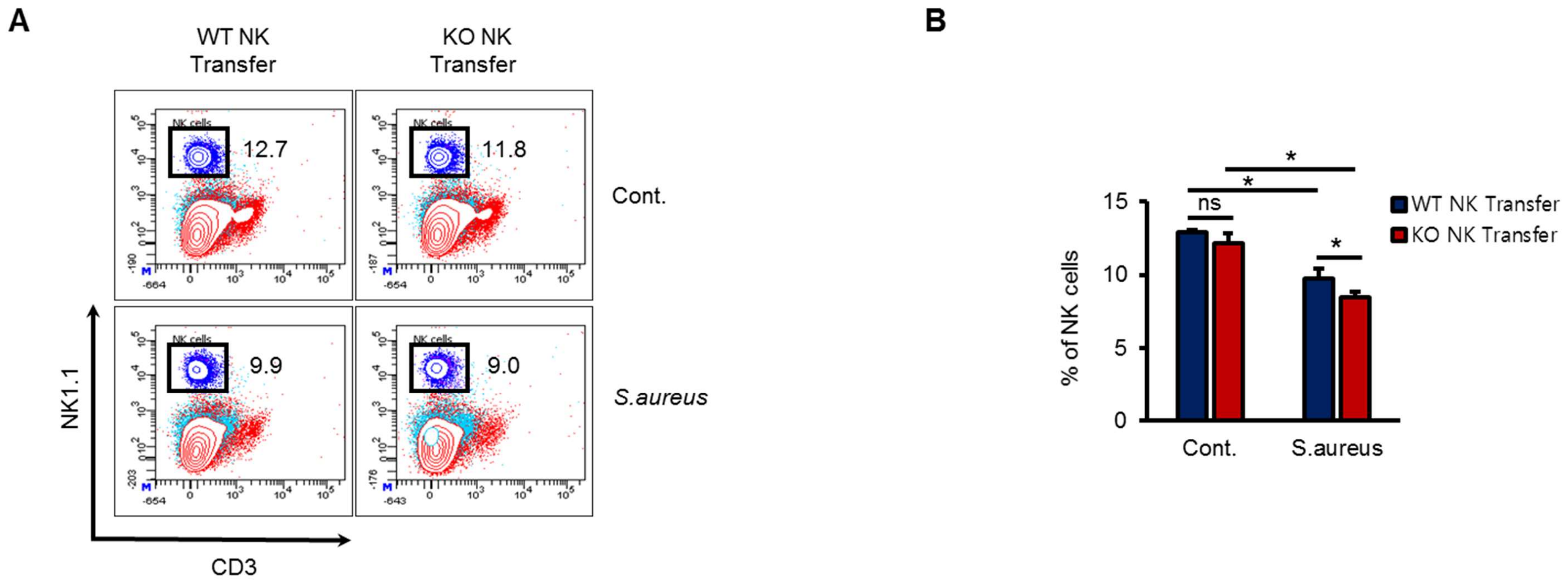

Figure S6. WT or KO NK cells were adoptive-transferred into Rag2$^{-/-}$ x Il2rg$^{-/-}$ mice for *S. aureus* infection study. **(A)** Representative dot plots of NK (CD3^{-} NK1.1^{+}) cells in mice spleen at 24h after infection. **(B)** Frequency of NK (CD3^{-}NK1.1^{+}) cells in mice spleen. The experiment was independently repeated at least three times and data are mean ± SD (n=5). Statistical significance was determined using Student's t tests. *$p < 0.05$, ns (not significant).
